# Evaluation of hydrolyzed corn steep liquor as an alternative feed ingredient in broiler nutrition: impacts on performance, litter quality, gut integrity, and bone mineralization

**DOI:** 10.1016/j.psj.2026.107548

**Published:** 2026-08-01

**Authors:** Hosna Hajati, AmirHossein Alizadeh Ghamsari, Seyyad Abdollah Hosseini

**Affiliations:** Department of Animal and Poultry Nutrition and Physiology, Animal Science Research Institute of Iran, Agricultural Research, Education and Extension Organization (AREEO), Karaj, Iran

**Keywords:** Hydrolyzed corn steep liquor, Digestibility, Bone quality, Footpad dermatitis, Litter quality

## Abstract

The present study was conducted to evaluate the effects of dietary incorporation with hydrolyzed corn steep liquor (**HCSL**) on growth performance, tibia characteristics, apparent digestibility of nutrients, litter quality, footpad dermatitis, jejunum morphology and ileal microbial population. A total of 750 one-day-old male and female broilers (Ross 308) were allocated to five dietary treatments with six replicates of 25 birds each in a completely randomized design. The experimental treatments included a basal corn–soybean meal diet without HCSL (HCSL-0) and the same basal diet supplemented with 2.5, 5, 7.5, or 10% HCSL. Data were analyzed using the GLM procedure of SAS, treatment means were compared by Tukey’s test, and orthogonal polynomial contrasts were applied to evaluate linear and quadratic responses to increasing dietary HCSL levels. During 25 to 42 days of age, daily feed intake decreased from 2654.39 g in the control to 2271.47 g in the HCSL-10 group (P = 0.004), with a significant linear effect (P < 0.001). Cumulative body weight gain declined from 2346.69 g in the control to 1672.14 g in the HCSL-10 treatment (P = 0.004), while feed conversion ratio increased from 1.56 to 1.91 (P = 0.003). Crude protein digestibility was significantly reduced in HCSL-10 treatment (55.52%) compared to HCSL-0 (64.54%), HCSL-2.5 (63.56%), and HCSL-5 (63.79%) treatments (P = 0.008). Bone weight, bone length, and the weight to length index of the bone in the HCSL-2.5 treatment were significantly greater than those in the HCSL-7.5 and HCSL-10 treatments (P < 0.05). Moreover, diameter of the medullary canal and bone strength in the control treatment was higher than in the HCSL-10 treatment (P < 0.05). The use of HCSL-10 relative to the control and HCSL-2.5 treatments resulted in an increase in litter ammoniacal nitrogen, litter pH and footpad dermatitis scoring. The HCSL-10 treatment, compared with control, HCSL-2.5 and HCSL-5 treatments, decreased villus height, villus surface area, and *Lactobacillus* population, while increasing *Escherichia coli* and coliform populations (P < 0.05). In conclusion, low dietary levels of HCSL, particularly 2.5%, could be used as a potential alternative feed ingredient in broiler diets without adverse effects, whereas high inclusion levels, especially 10%, negatively affected growth performance, nutrient digestibility, intestinal health, bone quality, litter condition, and microbial balance.

## Introduction

Feed expenses constitute nearly 70% of the total costs in commercial broiler production systems (Mallick et al., 2020). With the ongoing intensification of global poultry production, stable high-quality protein supplies are critical to sustaining efficient industrial systems (Kim et al., 2019). Soybean meal (**SBM**), due to its favorable amino acid profile and high nutrient digestibility, has traditionally been the primary protein source in broiler diets (Ma et al., 2024; Zhang et al., 2025). Nonetheless, recent instability in SBM prices (stemming from fluctuations in global grain production, changes in trade policies, and increasing energy costs) has significantly increased feed expenses and raised concerns about the long‑term sustainability of the poultry sector (Swick, 2011; Zhuang et al., 2025). As a result, the search for affordable and nutritionally sound substitutes for SBM has emerged as a key focus in animal nutrition research (Abasubong et al., 2025). Corn steep liquor (**CSL**), a by‑product of the corn wet‑milling industry, contains substantial levels of protein, short peptides, free amino acids, fermentable sugars, vitamins, trace minerals, and organic acids making it a viable alternative protein ingredient (Zhang et al., 2021). Global annual CSL production is ∼4.3–4.8 million dry tons, based on starch output (∼88–98 million tons/year, 75% corn) and wet-milling mass balance (Vilpoux and Junior, 2023; Zhang et al., 2021). Notably, CSL is economically advantageous and its utilization not only lowers feed costs but also contributes to reducing environmental burdens associated with industrial by‑products, thereby offering a sustainable alternative to SBM (Guo et al., 2026). Beyond its role as a nutrient source, CSL may also exert functional effects on gut health and metabolic efficiency (Hamidifard et al., 2023). More broadly, the growing interest in nontraditional feedstuffs in poultry nutrition reflects the need to partially replace conventional ingredients such as soybean meal and corn with alternative, nutritionally and economically feasible resources (Alshelmani et al., 2021).

Several studies have reported beneficial effects of moderate CSL incorporation on broiler performance, potentially due to improved nutrient digestibility and the beneficial effects of fermentation-derived organic acids on gut environment (Ebrahimpour et al., 2022; Hamidifard et al., 2023). Nevertheless, high conventional CSL inclusion often causes adverse physiological effects, limiting its practical use (Lee et al., 2025). Dietary inclusion exceeding 10% may suppress the development of immune and digestive organs, and induce crop mucosal hyperplasia, and intestinal inflammation (Ullah et al., 2018). In the study by Tauqir et al. (2022), although the 3% SCL level improved performance compared to the control treatment, the use of 9 and 12% levels significantly reduced growth performance and nutrient digestibility. Likewise, findings with other corn-derived by-products such as corn distillers' dried grains with soluble indicate that moderate inclusion levels may be beneficial, whereas excessive inclusion can impair broiler performance; for example, Kairalla et al. (2025) reported that corn distillers' dried grains with soluble could be included up to 20% without adverse effects, while 30% reduced performance and carcass yield. Moreover, liquid CSL is limited to use as an acidic feed additive at very low doses (1–2 cc/kg of diet) and cannot function as a protein ingredient capable of replacing SBM (Hamidifard et al., 2023). These inherent drawbacks and application constraints of conventional CSL significantly restrict its potential for large scale adoption as a primary protein source in broiler nutrition. In the present study, a novel CSL product (hydrolyzed corn steep liquor: **HCSL**) was used in place of traditional CSL to assess its suitability as an SBM substitute.

Enzymatic hydrolysis may provide an effective strategy to overcome these limitations and improve the nutritional quality of CSL (Guo et al., 2026). Hydrolysis can degrade anti-nutritional compounds, reduce the molecular complexity of nutrients, and increase the availability of amino acids, peptides, and phosphorus for intestinal absorption (Wang et al., 2024). In addition, enzymatic treatment may reduce the negative effects associated with high acidity and indigestible compounds while enhancing the functional properties of CSL in the gastrointestinal tract (Idowu and Yupanqui, 2025). Improved nutrient digestibility, particularly protein and minerals utilization, may subsequently contribute to better skeletal development, and litter quality in rapidly growing broiler chickens. Nevertheless, limited information is available regarding the effects of HCSL on growth performance, bone quality, digestibility, litter quality, and gut health in broiler chickens. Therefore, the present study was conducted to evaluate the effects of dietary incorporation with HCSL on growth performance, tibia characteristics, apparent digestibility of protein and phosphorus, litter quality, footpad condition, jejunum morphology and ileal microbial population. It was hypothesized that enzymatic hydrolysis could reduce the adverse effects associated with high levels of CSL and improve its nutritional efficacy through enhanced nutrient availability and digestibility.

## Materials and methods

All of the animal experimentation procedures used in the experiment were approved by the Animal Care Committee and Animal Research Ethics Board, Animal Science Research Institute of Iran, Agricultural Research, Education and Extension Organization (AREEO) (Approval No:14-13-13-052-040332) and were in compliance with the European Union guidelines for the care and use of animals in research European Parliament.

### Housing, experimental design, and diet

In this study, a total of 750 one‑day‑old male and female broiler chicks (Ross 308 strain) were used. The average body weight of the chicks at day one was 43.16 ± 0.51 g. The experiment was conducted using a completely randomized design consisting of five treatments. Each treatment included six replicates, and 25 chicks were randomly assigned to each replicate (13 males and 12 females). The experimental treatments included: (1) a basal diet based on corn and soybean meal without hydrolyzed corn steep liquor (HCSL-0); (2) the basal diet supplemented with 2.5% hydrolyzed corn steep liquor (HCSL-2.5); (3) the basal diet supplemented with 5% hydrolyzed corn steep liquor (HCSL-5); (4) the basal diet supplemented with 7.5% hydrolyzed corn steep liquor (HCSL-7.5); and (5) the basal diet supplemented with 10% hydrolyzed corn steep liquor (HCSL-10). All diets were formulated according to the nutritional recommendations provided in the Ross 308 and were balanced using UFFDA ration formulation software. The formulated diets used during the starter (1–10 days), grower (11–24 days), and finisher (25–42 days) phases are presented in Tables 1,2,3. Birds were reared in floor pens measuring 100 cm × 70 cm × 60 cm (length × width × height). The lighting schedule was set at 23 h of light and 1 h of darkness during the first week of age, after which it was adjusted to 18 h light and 6 h darkness for the remainder of the experimental period. Relative humidity was maintained at approximately 50% throughout the trial. At chick placement, the house temperature was maintained at approximately 32 °C and gradually reduced each week until reaching 22 °C by the fourth week of age, after which it remained constant for the rest of the rearing period. Feed and water were provided ad libitum throughout the study. The vaccination program was carried out according to standard commercial broiler management practices. Briefly, birds were vaccinated against infectious bronchitis (H120) on day 1 via spray application; Newcastle disease and avian influenza (H_9_N_2_, Ulster 2C) on day 7 through ocular and subcutaneous administration; Newcastle disease (B1 and LaSota strains) on days 12, 19, and 26 via drinking water; and infectious bursal disease (Gumboro, D78 strain) on days 16 and 23 through drinking water.

**Table 1:** Ingredient composition and chemical composition of broiler diets during 1–10 days of age.

Ingredients (%)	Control (HCSL-0)	HCSL-2.5	HCSL-5	HCSL-7.5	HCSL-10
Corn grain	55.88	56.12	56.13	55.96	52.10
Soybean meal	34.51	32.06	29.64	27.25	28.17
Corn gluten meal	3.00	3.00	3.00	3.00	3.00
Hydrolyzed CSL	-	2.50	5.00	7.50	10.00
Soybean oil	2.60	2.49	2.46	2.92	3.13
Salt	0.31	0.26	0.22	0.17	0.12
Sodium bicarbonate	0.15	0.14	0.12	0.11	0.10
Calcium carbonate	1.25	1.66	1.83	1.85	1.90
Dicalcium phosphate	1.17	0.60	0.37	0.34	0.29
Methionine	0.23	0.23	0.23	0.23	0.22
Lysine	0.17	0.23	0.28	0.33	0.28
Threonine	0.21	0.21	0.21	0.21	0.17
Multi-enzyme	0.01	0.01	0.01	0.01	0.01
Probiotic	0.02	0.02	0.02	0.02	0.02
Vitamin–mineral premix	0.50	0.50	0.50	0.50	0.50
**Nutrient levels**					
ME (kcal/kg)	2975	2975	2975	2975	2975
Crude protein (%)	22.00	22.00	22.00	22.00	22.00
Dig. lysine (%)	1.26	1.26	1.26	1.26	1.26
Dig. methionine (%)	0.53	0.53	0.53	0.53	0.53
Dig. methionine + cystine (%)	0.95	0.95	0.95	0.95	0.95
Dig. threonine (%)	0.84	0.84	0.84	0.84	0.84
Calcium (%)	0.95	0.95	0.95	0.95	0.95
Available phosphorus (%)	0.50	0.50	0.50	0.50	0.50
Sodium (%)	0.18	0.18	0.18	0.18	0.18
**Analyzed value**					
Gross energy (kcal/kg)	4252	4248	4263	4256	4260
Crude protein (%)	22.01	21.98	22.02	22.00	22.01
Calcium (%)	0.94	0.94	0.95	0.96	0.95
Total phosphorus (%)	0.73	0.72	0.73	0.74	0.72

The premix used in the diet supplied the following per kilogram of diet: Vitamin A, 11,000 IU; Vitamin D_3_, 1,800 IU; Vitamin E, 36 mg; Vitamin K_3_, 5 mg; Vitamin B_12_, 1.6 mg; Thiamine, 1.53 mg; Riboflavin, 7.5 mg; Niacin, 30 mg; Pyridoxine, 1.53 mg; Biotin, 0.03 mg; Folic acid, 1 mg; Pantothenic acid, 12.24 mg; and Ethoxyquin, 0.125 mg.

Mineral contents included: Iron, 250 mg; Zinc sulfate, 84 mg; Manganese sulfate, 160 mg; Iodine, 1.6 mg; Copper sulfate, 20 mg; Selenium, 0.2 mg; and Cobalt, 0.4 mg.

**Table 2:** Ingredient composition and chemical composition of broiler diets during 11–24 days of age.

Ingredients (%)	Control (HCSL-0)	HCSL-2.5	HCSL-5	HCSL-7.5	HCSL-10
Corn grain	58.15	58.39	58.63	58.79	58.65
Soybean meal	34.95	32.49	30.03	27.59	25.20
Hydrolyzed CSL	-	2.50	5.00	7.50	10.00
Soybean oil	3.46	3.35	3.24	3.15	3.17
Salt	0.31	0.26	0.22	0.17	0.12
Sodium bicarbonate	0.15	0.14	0.12	0.11	0.10
Calcium carbonate	0.51	0.91	1.32	1.65	1.67
Dicalcium phosphate	1.61	1.04	0.47	-	-
Methionine	0.21	0.21	0.21	0.21	0.21
Lysine	0.02	0.07	0.13	0.18	0.23
Threonine	0.13	0.13	0.13	0.13	0.13
Multi-enzyme	0.01	0.01	0.01	0.01	0.01
Probiotic	0.02	0.02	0.02	0.02	0.02
Vitamin–mineral premix	0.50	0.50	0.50	0.50	0.50
**Nutrient levels**					
ME (kcal/kg)	3050	3050	3050	3050	3050
Crude protein (%)	20.50	20.50	20.50	20.50	20.50
Dig. lysine (%)	1.14	1.14	1.14	1.14	1.14
Dig. methionine (%)	0.49	0.49	0.49	0.49	0.49
Dig. methionine + cystine (%)	0.89	0.89	0.89	0.89	0.89
Dig. threonine (%)	0.76	0.76	0.76	0.76	0.76
Calcium (%)	0.75	0.75	0.75	0.75	0.75
Available phosphorus (%)	0.42	0.42	0.42	0.42	0.42
Sodium (%)	0.18	0.18	0.18	0.18	0.18
**Analyzed value**					
Gross energy (kcal/kg)	4368	4365	4357	4369	4371
Crude protein (%)	20.52	20.49	20.50	20.48	20.51
Calcium (%)	0.75	0.74	0.76	0.76	0.74
Total phosphorus (%)	0.68	0.67	0.68	0.66	0.67

The premix used in the diet supplied the following per kilogram of diet: Vitamin A, 11,000 IU; Vitamin D_3_, 1,800 IU; Vitamin E, 36 mg; Vitamin K_3_, 5 mg; Vitamin B_12_, 1.6 mg; Thiamine, 1.53 mg; Riboflavin, 7.5 mg; Niacin, 30 mg; Pyridoxine, 1.53 mg; Biotin, 0.03 mg; Folic acid, 1 mg; Pantothenic acid, 12.24 mg; and Ethoxyquin, 0.125 mg.

Mineral contents included: Iron, 250 mg; Zinc sulfate, 84 mg; Manganese sulfate, 160 mg; Iodine, 1.6 mg; Copper sulfate, 20 mg; Selenium, 0.2 mg; and Cobalt, 0.4 mg.

**Table 3:** Ingredient composition and chemical composition of broiler diets during 25–42 days of age.

Ingredients (%)	Control (HCSL-0)	HCSL-2.5	HCSL-5	HCSL-7.5	HCSL-10
Corn grain	65.57	65.81	66.05	66.25	64.92
Soybean meal	27.82	25.36	22.90	20.45	19.10
Hydrolyzed CSL	-	2.50	5.00	7.50	10.00
Soybean oil	3.03	2.92	2.81	2.71	2.93
Salt	0.31	0.26	0.22	0.17	0.12
Sodium bicarbonate	0.15	0.14	0.13	0.12	0.10
Calcium carbonate	0.52	0.93	1.33	1.71	1.72
Dicalcium phosphate	1.67	1.10	0.53	-	-
Methionine	0.19	0.19	0.19	0.19	0.19
Lysine	0.10	0.15	0.21	0.26	0.28
Threonine	0.13	0.13	0.13	0.13	0.13
Multi-enzyme	0.01	0.01	0.01	0.01	0.01
Probiotic	0.02	0.02	0.02	0.02	0.02
Vitamin–mineral premix	0.50	0.50	0.50	0.50	0.50
**Nutrient levels**					
ME (kcal/kg)	3100	3100	3100	3100	3100
Crude protein (%)	18.00	18.00	18.00	18.00	18.00
Dig. lysine (%)	1.02	1.02	1.02	1.02	1.02
Dig. methionine (%)	0.45	0.45	0.45	0.45	0.45
Dig. methionine + cystine (%)	0.82	0.82	0.82	0.82	0.82
Dig. threonine (%)	0.68	0.68	0.68	0.68	0.68
Calcium (%)	0.65	0.65	0.65	0.65	0.65
Available phosphorus (%)	0.36	0.36	0.36	0.36	0.36
Sodium (%)	0.68	0.68	0.68	0.68	0.68
**Analyzed value**					
Gross energy (kcal/kg)	4476	4482	4468	4472	4470
Crude protein (%)	17.98	17.99	18.02	18.00	18.01
Calcium (%)	0.67	0.65	0.66	0.64	0.66
Total phosphorus (%)	0.58	0.60	0.59	0.61	0.60

The premix used in the diet supplied the following per kilogram of diet: Vitamin A, 11,000 IU; Vitamin D_3_, 1,800 IU; Vitamin E, 36 mg; Vitamin K_3_, 5 mg; Vitamin B_12_, 1.6 mg; Thiamine, 1.53 mg; Riboflavin, 7.5 mg; Niacin, 30 mg; Pyridoxine, 1.53 mg; Biotin, 0.03 mg; Folic acid, 1 mg; Pantothenic acid, 12.24 mg; and Ethoxyquin, 0.125 mg.

Mineral contents included: Iron, 250 mg; Zinc sulfate, 84 mg; Manganese sulfate, 160 mg; Iodine, 1.6 mg; Copper sulfate, 20 mg; Selenium, 0.2 mg; and Cobalt, 0.4 mg.

### Nutritional ingredients

The chemical composition of HCSL was measured using the AOAC (2006) recommended method. Its amino acid profile was also determined by near-infrared reflectance spectroscopy (NIRS) by Radin Feedar Farda Company (Table 4).

**Table 4:** Chemical analysis and amino acid of hydrolyzed corn steep liquor (n = 10).

Chemical composition	Amount	Amino acid	Free amino acids	Hydrolyzed amino acids
Dry Matter (%)	96.60	Lysine	2.4	3.3
Protein (%)	40.50	Methionine	3.1	2.1
Fat (%)	0.10	Arginine	16.5	16.2
Fiber (%)	0.66	Threonine	<0.1	3.2
Total Phosphorus (%)	4.50	Valine	6.2	<0.1
Ash (%)	26.80	Alanine	13.0	14.2
Carbohydrates (%)	28.60	Isoleucine	3.3	3.4
Sodium (%)	0.76	Leucine	9.6	9.4
Potassium (%)	7.60	Phenylalanine	4.7	4.6
Manganese (mg/kg)	3626	Cysteine	0.5	4.4
Chlorine (%)	1.50	Glycine	3.8	7.6

#### Performance

Feed intake (**FI**) was recorded daily after weighing the feed provided to the birds. To determine the FI for each replicate, the amount of feed remaining at the end of each rearing period was subtracted from the total amount of feed offered during that period. Body weight gain (**BWG**) for each replicate in each experimental period was calculated by determining the difference between the final body weight and the body weight at the beginning of that period. On days 1, 10, 24, and 42 of age, all birds in each experimental unit were weighed collectively to determine body weight. The feed conversion ratio (**FCR**) was calculated separately for the starter, grower, and finisher phases. This parameter was obtained by dividing the average FI by the average BWG of the birds within each period. During the experimental period, mortality was monitored daily in each pen before feed distribution, and the weight of the dead birds was recorded. The daily mortality data were used to calculate the chicken‑day value for each experimental unit (Ebrahimi et al., 2026). Viability percentage = 100 - mortality percentage. European production index (EPE) = [Viability percentage × body weight (kg)] / [FCR (kg feed per kg gain) × age (day)] (Al-Garadi et al., 2026).

#### Digestibility assay

To estimate the apparent ileal digestibility of nutrients, titanium dioxide was incorporated into the experimental diets at a rate of 5 g/kg feed from day 39 onward and used as an indigestible external marker, following the procedure described by Nari and Ghasemi (2020). At the end of the trial (day 42), two male birds from each replicate were humanely euthanized by carbon dioxide inhalation, and ileal digesta samples were collected from the terminal ileum, specifically the segment extending from Meckel’s diverticulum to the ileo-cecal-colonic junction. The digesta were gently flushed with distilled water and transferred into plastic containers for subsequent analyses. Feed and ileal digesta samples were dried in a forced-air oven at 60 °C for 72 h and then kept at room conditions for an additional 24 h to allow moisture equilibration. Thereafter, all samples were finely ground to pass through a 0.75-mm screen using a laboratory mill (ZM 100, Retsch GmbH, Haan, Germany). Crude protein concentration was determined based on nitrogen content measured by the micro-Kjeldahl method and calculated using a conversion factor of 6.25. For mineral analyses, diet and ileal digesta samples were first ashed in a muffle furnace at 600 °C for 16 h. Titanium concentration in diets and ileal digesta was then determined spectrophotometrically at 410 nm (Spectronic 21D, Milton Roy Co., Rochester, NY, USA) after wet digestion with sulfuric acid and hydrogen peroxide, according to the method of Short et al. (1996). For calcium determination, ash samples were digested using nitric and perchloric acids, and calcium concentration was quantified by flame atomic absorption spectrophotometry (Varian Spectra AA 220FS, Varian Australia Pty Ltd, Victoria, Australia). Phosphorus concentration was measured after acid digestion by developing a phospho-molybdenum complex through the addition of acid molybdate and Fiske–SubbaRow reducer solution, and the resulting color intensity was read spectrophotometrically at 630 nm.

Apparent nutrient digestibility coefficients were calculated using the index method described by Kong and Adeola (2014), according to the following equation:

Apparent nutrient digestibility (AND, %) = [1 – (T_i_ ÷ T_o_) × (X_o_ ÷ X_i_)] × 100

where apparent nutrient digestibility is expressed as a percentage for crude protein, ash, calcium, and phosphorus; T_i_ represents the titanium concentration in the diet (g/kg DM); T_o_ denotes the titanium concentration in the ileal digesta or excreta (g/kg DM); and X_i_ and X_o_ indicate the nutrient concentrations in the diet and digesta/excreta, respectively.

#### Tibial bone characteristics

On the final day of the experiment, both the left and right tibiotarsi were harvested from two male birds from each replicate. To facilitate the removal of soft tissue, the bones were labeled, placed in plastic bags, and immersed in boiling water (100 °C) for 10 minutes. After cooling to room temperature, the bones were manually defleshed, the patella was removed, and the samples were air-dried for 24 hours. The weight, length, and diaphysis diameter (at the midpoint) of the tibiotarsi were recorded using a high-precision digital balance (0.01 g; Model K-E-B-602, Kerona, Shanghai, China) and a digital caliper (0.01 mm; Bakingwin, Shenzhen, China). The strength of the tibia of the left leg against pressure was measured in newtons per meter (Ebrahimi et al., 2025). Strength test was performed by a Santam device (Model DBBP-500, Santam Engineering Co., Tehran, Iran) at 60.00 mm per min. The resulting bone fragments were subsequently used for chemical analysis. For ash determination, bone fragments were dried in a forced-air oven at 150 °C for 24 hours and then incinerated in a muffle furnace at 600 °C for 6 hours. The ash content was calculated as a percentage of the dry bone weight (AOAC, 2006). Calcium concentration in tibia samples was quantified by flame atomic absorption spectrophotometry (Varian Spectr AA 50B Atomic Absorption Spectrometer; Varian Ltd., USA) in accordance with AOAC (2006) procedures. Tibial phosphorus content was also determined spectrophotometrically using a Jenway Genova MK3 spectrophotometer (Jenway, UK) following the analytical methods outlined by AOAC (2006). The contralateral tibiotarsus was cross-sectioned at the midpoint of the diaphysis. The thickness of the medial and lateral walls was measured using digital calipers and DinoCapture 2.0 software. The medullary canal diameter was calculated by subtracting the sum of the medial and lateral wall thicknesses from the total diaphysis diameter. To evaluate bone health and mineralization, the following indices were calculated (Çapar Akyüz et al., 2024): Weight-to-length index: Bone weight/ Bone length (An indicator of bone density).

$$Tibiotarsal\;index\;\left(\%\right)=\frac{Diaphysis\;diameter-Medullary\;canal\;diameter}{Diaphysis\;diameter}\times \;100$$

$$Bone\;strength\;index=\frac{\text{Tibial}\;\text{length}}{\sqrt[{3}]{\text{Tibial}}\text{weight}}$$

#### Litter quality assessment

Litter samples were collected from five representative locations within each pen, including two points at the front of the pen near the feeders and drinkers, two points at the rear area away from feeding and drinking equipment, and one point at the center of the pen (Brink et al., 2022a). On day 42, approximately 100 g of litter was obtained from each sampling location and combined to form a composite sample for each pen. The collected samples were dried in a forced‑air oven at 65 °C until constant weight to determine litter dry matter content. Crude protein content of litter samples was determined based on nitrogen concentration using the Kjeldahl method, and crude protein was calculated as nitrogen × 6.25 according to AOAC procedures. For pH determination, additional litter samples were collected from three locations within each pen, including the left and right rear areas and the central area. At each sampling point, 20 g of litter was mixed with 100 mL of distilled water and allowed to stand for 30 min to ensure proper equilibration. The pH of the fresh litter suspension was then measured using a portable digital pH meter. The three measurements obtained from each pen were averaged to calculate the mean litter pH for each pen and subsequently for each treatment (Brink et al., 2022a). Ammoniacal nitrogen (NH₃–N) in litter samples was determined according to the method of Stuch and Scafie (1991). Briefly, litter samples were extracted with 2 M KCl, shaken for 30–60 min, and filtered through Whatman No. 42 filter paper. The ammonium concentration in the filtrate was then quantified colorimetrically using Nessler reagent, and absorbance was measured spectrophotometrically at 425 nm. Ammoniacal nitrogen concentration was calculated using a standard curve prepared from ammonium sulfate solutions.

#### Footpad dermatitis scoring

Footpad condition was evaluated at the end of the experiment using a visual scoring system commonly applied in broiler welfare assessments. The plantar surface of both feet of each bird was examined, and lesions were scored according to a five‑point scale. Score 0 indicated a completely healthy footpad with no visible lesions. Score 1 represented mild lesions affecting less than 10% of the footpad surface. Score 2 corresponded to lesions affecting less than 25% of the surface area. Score 3 indicated moderate lesions involving less than 50% of the footpad. Score 4 represented severe lesions affecting more than 50% of the footpad surface. Paws were scored by the same researchers in all treatments (Shepherd et al., 2017).

#### Intestinal morphology

At 42 days of age, two male birds from each replicate were selected, and a segment of the jejunum was collected for morphological assessment following the procedure described by Khalilzadeh et al. (2025). Approximately 1 cm of the jejunum was excised and gently rinsed with 0.9% saline solution to remove any intestinal contents. The collected tissue samples were then fixed in 10% buffered formalin to preserve their structure for subsequent histological examination. Following fixation, the samples underwent routine histological processing, including dehydration, clearing, and embedding in paraffin. The paraffin-embedded tissues were then sectioned at a thickness of 7 μm using a microtome (Microm, HM 335), and the resulting sections were mounted on glass slides. The prepared slides were first deparaffinized using xylene and subsequently rehydrated through a graded series of ethanol solutions. Afterward, the tissue sections were stained with hematoxylin and eosin (H&E) and examined using a light microscope. Morphological parameters evaluated in the intestinal samples included villus height, villus width, crypt depth and villus surface area. Villus height was determined by measuring the distance from the villus tip to the upper boundary of the lamina propria. Crypt depth was measured from the base of the villus-crypt junction to the tip of the muscularis mucosa. The ratio of villus height to crypt depth (VH/CD) was calculated by dividing villus height by crypt depth (Hassanlou et al., 2026). Additionally, the surface area of the villi was estimated using the formula (2π) × (villus width × 0.5) × villus length (Sakamoto et al., 2000). The villus is often considered to be a cylindrical structure and thus the villus absorptive surface area can be assessed through the listed procedure.

#### Specific ileal microbiota counts

The gastrointestinal tracts of 12 male birds per treatment were dissected, and ileal contents were aseptically collected into sterile containers and stored at −20 °C until analysis (days 42). Ileal digesta were sampled from the region extending from Meckel’s diverticulum to the ileo-cecal junction. Approximately 1 g of ileal content was suspended in 9 mL of sterile 0.9% saline solution and homogenized, followed by preparation of appropriate serial dilutions. *Lactobacillus* spp., *Coliform*, and *Escherichia coli* populations were enumerated using de Man, Rogosa and Sharpe (MRS) agar, sulfite iron agar (SIA), and MacConkey agar (Merck, Darmstadt, Germany), respectively. Microbial counts were expressed as log10 colony-forming units (CFU) per gram of ileal digesta (Althagafi et al., 2025).

#### Statistical analysis

All experimental data were initially entered and organized using Microsoft Excel. Calculations related to the measured traits, as well as data classification and preparation for statistical analysis, were performed in Excel. The collected data were statistically analyzed using the General Linear Model (GLM) procedure of SAS software (Version 9.2). For performance variables, the statistical model used was *Y_ij_* = *μ* +*T_i_* +*e_ij_*​. For the remaining variables, the model was *Y_ijk_* = *μ* + *T_i_* + *e_ij_* + *ε_ijk_*. In these models, *Y_ij_*​ and *Y_ijk_*​ denote the observed responses, *μ* represents the overall mean, *Ti*​ corresponds to the dietary treatment effect, *e_ij_*​ is the random error term, and *ε_ijk_* refers to the subsampling error. For growth performance traits, the pen was considered the experimental unit, whereas the individual bird was considered the experimental unit for ileal digestibility, tibial bone characteristics, intestinal morphology, and ileal microbiota. Differences among treatment means were evaluated using Tukey’s multiple range test. In addition to the comparison of means, orthogonal polynomial contrasts were used to determine the linear and quadratic effects of increasing levels of HCSL in the diets. Data expressed as percentages or ratios (non-continuous variables) were subjected to arcsine transformation prior to analysis to meet the assumptions of normality and homogeneity of variance.

## Results

### Broiler performance

In Table 5, the effect of different levels of HCSL on the performance indices of broiler chickens during the rearing period has been reported. Daily FI during the 25 to 42 days of age period in the HCSL-10 treatment was significantly lower than that of the other treatments (P = 0.004). Over the whole rearing period (1 to 42 days of age), a significant difference was also observed (P = 0.013), such that the control and HCSL-2.5 treatments had the highest FI, and the HCSL-10 treatment had the lowest amount. BWG during the 1 to 10, 25–42, and 1–42 days of age periods was significantly affected by different HCSL levels (P < 0.01), such that the control and HCSL-2.5 treatments showed greater weight gain compared with the HCSL-5, HCSL-7.5, and HCSL-10 treatments. During the 11 to 24 days of age period, the greatest weight gain was recorded in the HCSL-2.5 treatment and the lowest amount in the HCSL-10 treatment (P = 0.001). FCR during all rearing periods was significantly affected by HCSL levels, and the use of HCSL-10 compared with the HCSL-0, HCSL-2.5, and HCSL-5 levels caused an increase in FCR (P < 0.05). The EPI decreased significantly with increasing dietary HCSL levels, with the highest value in the control group and the lowest at 10% HCSL (P < 0.001).

**Table 5:** Effect of different levels of hydrolyzed corn steep liquor on growth performance of broiler chickens (n = 150).

Items	Control (HCSL-0)	HCSL-2.5	HCSL-5	HCSL-7.5	HCSL-10	SEM	*P*-value		Orthogonal polynomials	
									Linear	Quadratic
**Average feed intake, g/d**										
1-10d	192.02	197.55	179.67	185.87	172.63	7.20	0.147		0.068	0.618
11-24d	831.47	888.43	841.79	801.38	763.22	31.16	0.092		0.055	0.117
25-42d	2654.39^a^	2638.69^a^	2484.41^a^	2478.07^a^	2271.47^b^	69.33	0.004		0.001	0.369
1-42d	3677.88^a^	3724.67^a^	3505.87^ab^	3465.32^ab^	3207.32^b^	94.52	0.013		0.001	0.225
**Body weight gain, g/d**										
1-10d	174.64^a^	179.33^a^	158.97^ab^	149.31^b^	135.05^b^	7.83	0.002		0.001	0.355
11-24d	643.53^ab^	657.65^a^	590.42^b^	525.67^c^	476.53^c^	19.15	0.001		0.001	0.086
25-42d	1528.52^a^	1477.22^a^	1334.61^b^	1240.56^b^	1060.56^c^	44.66	0.001		0.001	0.213
1-42d	2346.69^a^	2314.20^a^	2084.00^b^	1915.54^b^	1672.14^c^	61.74	0.004		0.001	0.121
**Feed conversion ratio**										
1-10d	1.10^c^	1.11^c^	1.13^bc^	1.24^ab^	1.29^a^	0.04	0.011		0.001	0.258
11-24d	1.29^d^	1.35^cd^	1.42^bc^	1.52^ab^	1.60^a^	0.09	0.001		0.006	0.836
25-42d	1.73^d^	1.79^d^	1.86^c^	1.99^b^	2.14^a^	0.05	0.001		0.001	0.202
1-42d	1.56^b^	1.60^b^	1.68^b^	1.80^ab^	1.91^a^	0.06	0.003		0.001	0.424
Mortality, %	2.00	3.66	2.00	3.33	3.66	0.62	0.197		0.129	0.887
EPI	3569.84^a^	3375.05^a^	2950.00^ab^	2500.56^b^	2056.19^c^	63.86	0.001		0.001	0.030

The experimental treatments included: The experimental treatments included: (1) a basal diet based on corn and soybean meal without corn steep liquor (HCSL-0); (2) the basal diet supplemented with 2.5% corn steep liquor (HCSL-2.5); (3) the basal diet supplemented with 5% corn steep liquor (HCSL-5); (4) the basal diet supplemented with 7.5% corn steep liquor (HCSL-7.5); and (5) the basal diet supplemented with 10% corn steep liquor (HCSL-10). SEM: standard error of means. EPI: European Production Index.

^a-b^ Means within a variable with no common superscript differ significantly (P < 0.05).

### Nutrient digestibility

The effect of different levels of HCSL on nutrient digestibility in broiler diets is shown in Table 6. Different levels of HCSL had a significant effect on crude protein digestibility of the diet, and the use of HCSL-10, in comparison with the control, HCSL-2.5, and HCSL-5 treatments, resulted in a reduction in crude protein digestibility (P = 0.008). The digestibility of ash, calcium, and phosphorus was not affected by the experimental treatments, and no significant differences were observed among the groups (P > 0.05).

**Table 6:** Effect of different levels of hydrolyzed corn steep liquor on nutrients digestibility (%) in broiler diets (n = 12).

Items	Control (HCSL-0)	HCSL-2.5	HCSL-5	HCSL-7.5	HCSL-10	SEM	*P*-value		Orthogonal polynomials	
									Linear	Quadratic
Crude protein	64.54^a^	63.56^a^	63.79^a^	60.17^ab^	55.52^b^	2.58	0.008		0.011	0.251
Ash	45.10	48.37	40.36	41.38	43.55	4.32	0.576		0.463	0.674
Calcium	67.34	71.92	73.56	71.12	62.56	5.03	0.459		0.518	0.112
Phosphorus	35.45	36.42	36.20	39.81	38.47	2.58	0.791		0.751	0.432

The experimental treatments included: The experimental treatments included: (1) a basal diet based on corn and soybean meal without corn steep liquor (HCSL-0); (2) the basal diet supplemented with 2.5% corn steep liquor (HCSL-2.5); (3) the basal diet supplemented with 5% corn steep liquor (HCSL-5); (4) the basal diet supplemented with 7.5% corn steep liquor (HCSL-7.5); and (5) the basal diet supplemented with 10% corn steep liquor (HCSL-10). SEM: standard error of means.

^a-b^ Means within a variable with no common superscript differ significantly (P < 0.05).

### Bone quality

In Table 7, the effect of different levels of HCSL on the physical and chemical characteristics of the tibia bone of broiler chickens is reported. Bone weight, bone length, and the weight-to-length index of the bone in the HCSL-2.5 treatment were significantly greater than those in the HCSL-7.5 and HCSL-10 treatments (P < 0.05). The diameter of the medullary canal also showed a significant difference among treatments (P = 0.020), and the control treatment had a larger diameter compared with the HCSL-10 treatment. Moreover, bone strength in the control treatment was higher than in the HCSL-10 treatment (P = 0.034). Regarding the chemical characteristics, the bone ash percentage was affected by the treatments (P = 0.017), such that the use of HCSL-10 compared with the control, HCSL-2.5, HCSL-5, and HCSL-7.5 treatments resulted in a reduction in bone ash.

**Table 7:** Effect of different levels of hydrolyzed corn steep liquor on tibia bone quality of broiler chickens (n = 12).

Items	Control (HCSL-0)	HCSL-2.5	HCSL-5	HCSL-7.5	HCSL-10	SEM	*P*-value		Orthogonal polynomials	
									Linear	Quadratic
**Physical characteristics**										
Weight (g)	6.92^ab^	7.58^a^	7.00^ab^	5.93^b^	5.92^b^	0.23	0.012		0.002	0.247
Length (mm)	99.70^ab^	102.26^a^	99.43^ab^	97.02^bc^	93.74^c^	0.74	0.004		0.001	0.163
Diaphysis diameter (mm)	8.54	8.39	8.38	7.64	7.84	0.16	0.077		0.019	0.397
Medullary canal diameter (mm)	4.61^a^	4.29^ab^	4.11^ab^	3.62^ab^	3.48^b^	0.20	0.020		0.001	0.066
Mid-wall thickness (mm)	1.96	2.05	2.13	2.08	2.11	0.09	0.943		0.883	0.577
Weight/length index	69.15^ab^	74.13^a^	70.43^ab^	61.07^b^	63.10^b^	2.04	0.048		0.004	0.170
Strength (N)	4878.7^a^	4055.7^ab^	4296.0^ab^	3535.0^ab^	3074.0^b^	72.29	0.034		0.001	0.151
Robusticity index	5.28	5.25	5.23	5.40	5.23	0.32	0.143		0.711	0.947
Tibiotarsal index	46.38	49.03	50.86	54.24	53.59	3.28	0.531		0.227	0.828
**Chemical characteristics**										
Ash (%)	41.76^a^	41.93^a^	42.93^a^	42.03^a^	39.50^b^	0.43	0.017		0.004	0.210
Calcium (%)	15.54	15.57	15.76	15.68	14.39	0.25	0.386		0.176	0.119
Phosphorus (%)	5.55	5.57	5.53	5.52	5.31	0.14	0.568		0.233	0.548

The experimental treatments included: The experimental treatments included: (1) a basal diet based on corn and soybean meal without corn steep liquor (HCSL-0); (2) the basal diet supplemented with 2.5% corn steep liquor (HCSL-2.5); (3) the basal diet supplemented with 5% corn steep liquor (HCSL-5); (4) the basal diet supplemented with 7.5% corn steep liquor (HCSL-7.5); and (5) the basal diet supplemented with 10% corn steep liquor (HCSL-10). SEM: standard error of means.

^a-b^ Means within a variable with no common superscript differ significantly (P < 0.05).

### Litter quality

Table 8 shows the changes in the litter quality of broiler chickens at 42 days of age in response to different levels of HCSL. The results showed that litter pH was affected by the experimental treatments (P = 0.036), such that adding HCSL-10 in comparison with the HCSL-2.5 treatment caused an increase in litter pH. Also, the amount of litter ammoniacal nitrogen significantly changed (P = 0.027), and the use of HCSL-10 relative to the control and HCSL-2.5 treatments resulted in an increase in ammoniacal nitrogen. Litter dry matter and crude protein were not affected by the experimental treatments, and no significant difference was observed among the groups (P > 0.05).

**Table 8:** Effect of different levels of hydrolyzed corn steep liquor on litter quality at 42 days of age (n = 12).

Items	Control (HCSL-0)	HCSL-2.5	HCSL-5	HCSL-7.5	HCSL-10	SEM	*P*-value		Orthogonal polynomials	
									Linear	Quadratic
Dry matter (%)	85.40	82.26	85.40	81.70	85.49	1.39	0.482		0.931	0.183
Crude protein (%)	6.10	6.09	6.53	6.55	7.45	0.69	0.861		0.153	0.590
pH	6.32^bc^	6.23^c^	6.46^ab^	6.61^ab^	6.70^a^	0.06	0.036		0.001	0.218
Ammoniacal nitrogen (mg/100 g)	64.14^c^	65.77^c^	74.10^b^	74.28^b^	83.46^a^	2.49	0.027		0.001	0.459

The experimental treatments included: The experimental treatments included: (1) a basal diet based on corn and soybean meal without corn steep liquor (HCSL-0); (2) the basal diet supplemented with 2.5% corn steep liquor (HCSL-2.5); (3) the basal diet supplemented with 5% corn steep liquor (HCSL-5); (4) the basal diet supplemented with 7.5% corn steep liquor (HCSL-7.5); and (5) the basal diet supplemented with 10% corn steep liquor (HCSL-10). SEM: standard error of means.

^a-b^ Means within a variable with no common superscript differ significantly (P < 0.05).

### Footpad dermatitis scoring

In Table 9, the effect of different levels of HCSL on footpad dermatitis score at 42 days of age is presented. The results showed that the frequency of score zero was affected by the treatments (P = 0.001), and the use of HCSL-5, HCSL-7.5, and HCSL10, in comparison with the control and HCSL-2.5 treatments, caused a reduction in the frequency of this score. Also, the frequency of score 1 increased with the addition of HCSL-5, HCSL-7.5, and HCSL-10 compared with the control and HCSL-2.5 treatments (P = 0.019). The frequency of score 3 was also affected by the treatments (P = 0.005), such that the use of HCSL-10, compared with the control and HCSL-2.5 treatments, resulted in an increase in this score. In addition, the use of HCSL-7.5 and HCSL-10, in comparison with the control, HCSL-2.5, and HCSL-5 treatments, caused an increase in the frequency of score 4 (P = 0.001).

**Table 9:** Effect of different levels of hydrolyzed corn steep liquor on footpad dermatitis scoring at 42 days of age (n = 12).

Items	Control (HCSL-0)	HCSL-2.5	HCSL-5	HCSL-7.5	HCSL-10	SEM	*P*-value		Orthogonal polynomials	
									Linear	Quadratic
Score 0	100.0^a^	100.0^a^	85.0^b^	80.0^b^	70.0^c^	2.36	0.001		0.001	0.262
Score 1	0.0^b^	0.0^b^	5.0^a^	5.0^a^	5.0^a^	1.29	0.019		0.001	0.305
Score 2	0.0	0.0	5.0	5.0	5.0	1.58	0.075		0.004	0.401
Score 3	0.0^b^	0.0^b^	5.0^ab^	5.0^ab^	10.0^a^	1.70	0.005		0.001	0.435
Score 4	0.0^c^	0.0^c^	0.0^c^	5.0^b^	10.0^a^	1.29	0.001		0.001	0.093

Score 0: Healthy; Score 1: Less than 10% of the footpad affected; Score 2: Less than 25% of the footpad affected; Score 3: Less than 50% of the footpad affected; Score 4: More than 50% of the footpad affected.

The experimental treatments included: The experimental treatments included: (1) a basal diet based on corn and soybean meal without corn steep liquor (HCSL-0); (2) the basal diet supplemented with 2.5% corn steep liquor (HCSL-2.5); (3) the basal diet supplemented with 5% corn steep liquor (HCSL-5); (4) the basal diet supplemented with 7.5% corn steep liquor (HCSL-7.5); and (5) the basal diet supplemented with 10% corn steep liquor (HCSL-10). SEM: standard error of means.

^a-b^ Means within a variable with no common superscript differ significantly (P < 0.05).

The provided images display the status of footpad dermatitis at different levels of HCSL (Fig. 1). In the images related to HCSL-0 and HCSL-2.5 treatments, the footpads have a healthy appearance, and no signs of lesions are visible. In the HCSL-5 treatment, mild signs of footpad damage are observed. With an increase in the HCSL level to 7.5 percent, the severity of lesions becomes visible, and dark areas on the footpad become apparent. In the HCSL-10 treatment as well, the lesions are wider and deeper, and more dark areas are seen compared to other treatments.

**Fig. 1:**
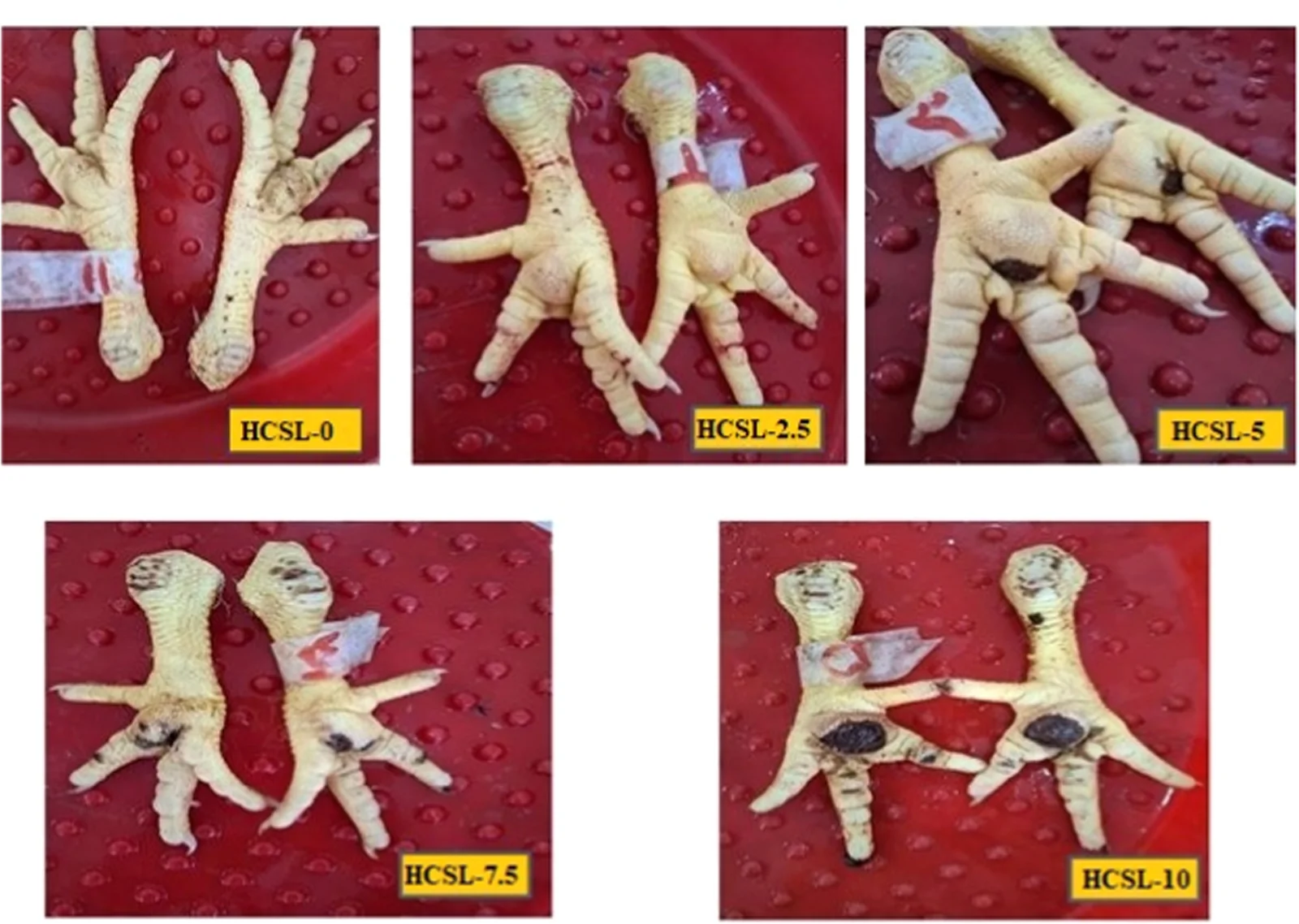
Effect of different levels of hydrolyzed corn steep liquor on footpad dermatitis scoring.

### Jejunum morphology

Table 10 indicates the effect of different levels of HCSL on the jejunal morphology of broiler chickens. Villus height was affected by the experimental treatments, such that the use of HCSL-2.5 compared with HCSL-10 resulted in an increase in villus height and the villus height-to-crypt depth ratio (P < 0.05). Also, the use of HCSL-10, in comparison with the control, HCSL-2.5, and HCSL-5 treatments, caused a reduction in villus surface area (P = 0.029).

**Table 10:** Effect of different levels of hydrolyzed corn steep liquor on jejunum morphology of broiler chickens (n = 12).

Items	Control (HCSL-0)	HCSL-2.5	HCSL-5	HCSL-7.5	HCSL-10	SEM	*P*-value		Orthogonal polynomials	
									Linear	Quadratic
Villus height (µm)	1085.8^ab^	1169.5^a^	1112.4^ab^	1060.3^ab^	1006.1^b^	20.53	0.041		0.001	0.001
Villus width (µm)	135.10	142.10	145.33	125.73	120.93	8.49	0.199		0.102	0.150
Crypt depth (µm)	170.30	156.90	165.33	175.43	180.57	7.70	0.151		0.114	0.184
Villus height /crypt depth	6.39^ab^	7.53^a^	6.74^ab^	6.04^ab^	5.61^b^	0.38	0.035		0.014	0.036
Villus surface area (µm²)	457698^b^	521971^a^	508016^a^	417046^bc^	383918^c^	905.6	0.029		0.001	0.001

The experimental treatments included: The experimental treatments included: (1) a basal diet based on corn and soybean meal without corn steep liquor (HCSL-0); (2) the basal diet supplemented with 2.5% corn steep liquor (HCSL-2.5); (3) the basal diet supplemented with 5% corn steep liquor (HCSL-5); (4) the basal diet supplemented with 7.5% corn steep liquor (HCSL-7.5); and (5) the basal diet supplemented with 10% corn steep liquor (HCSL-10). SEM: standard error of means.

^a-b^ Means within a variable with no common superscript differ significantly (P < 0.05).

### Ileal microbial population

In Table 11, the effect of different levels of HCSL on the microbial population in the ileum content of broiler chickens is reported. The population of *E. coli* was affected by the experimental treatments (P = 0.001), such that the use of HCSL-7.5 and HCSL-10, compared with the control, HCSL-2.5, and HCSL-5 treatments, resulted in an increase in the population of this bacteria. Also, the population of *coliforms* showed a significant difference among the treatments (P = 0.001), and the 2.5% and 5% HCSL treatments had a lower population of *coliforms* compared with the control, HCSL-7.5, and HCSL-10 treatments. The use of HCSL-7.5 and HCSL-10, compared with the control treatment, caused a decrease in the population of *Lactobacillus* (P = 0.001).

**Table 11:** Effect of different levels of corn steep liquor on the microbial population of ileal contents (log CFU per gram of content).

Items	Control (HCSL-0)	HCSL-2.5	HCSL-5	HCSL-7.5	HCSL-10	SEM	*P*-value		Orthogonal polynomials	
									Linear	Quadratic
*E. coli*	6.70^b^	6.51^c^	6.60^bc^	6.87^a^	6.97^a^	0.02	0.001		0.001	0.009
*Coliforms*	7.87^a^	7.51^b^	7.62^b^	7.96^a^	7.90^a^	0.03	0.001		0.001	0.041
*Lactobacillus*	8.21^a^	7.76^bc^	7.94^bc^	7.44^d^	7.65^cd^	0.05	0.001		0.001	0.028

The experimental treatments included: The experimental treatments included: (1) a basal diet based on corn and soybean meal without corn steep liquor (HCSL-0); (2) the basal diet supplemented with 2.5% corn steep liquor (HCSL-2.5); (3) the basal diet supplemented with 5% corn steep liquor (HCSL-5); (4) the basal diet supplemented with 7.5% corn steep liquor (HCSL-7.5); and (5) the basal diet supplemented with 10% corn steep liquor (HCSL-10). SEM: standard error of means.

^a-b^ Means within a variable with no common superscript differ significantly (P < 0.05).

## Discussion

### Performance

The present study demonstrated that birds fed the 2.5 and 5% HCSL diet showed performance comparable to, the control group, whereas inclusion of higher levels, particularly 10% HCSL, resulted in reduced FI, decreased BWG, and poorer FCR. These findings indicate that the response of broilers to HCSL is dose-dependent, and while low inclusion levels may be utilized in broiler diets without detrimental effects, excessive inclusion levels can negatively affect growth performance. In contrast to the findings of the present experiment, many previous studies reported that the inclusion of CSL up to approximately 10% had no adverse effects and could even improve growth performance or maintain performance comparable to the control diet. For example, Ebrahimpour et al. (2022) reported that adding 4% dried CSL increased FI and BWG in Ross 308 broilers during the first 10 days, which they attributed to improved palatability and possible beneficial effects of CSL-associated organic acids on gut health. Likewise, Ullah et al. (2018) observed that a 10% inclusion level of CSL produced the highest BWG and the most favorable FCR in Hubbard × Hubbard broilers, whereas increasing the level to 15% maintained BWG but did not provide additional improvements in feed efficiency. Yazdanyar et al. (2015) also found that CSL levels above 10% had detrimental effects on BWG, FI, and FCR, which may have been related to increased diet viscosity and elevated lactic acid concentrations associated with digestive discomfort. Conversely, moderate CSL inclusion (≤10%) enhanced growth performance, potentially through mechanisms such as improved nutrient digestibility and possible changes in the gastrointestinal environment. However, in agreement with the results of the present study, Tauqir et al. (2022) reported that the use of a mixture of CSL and enzyme (50:50 ratio) at levels of 9 and 12% reduced growth performance and FI, while the level of 3% showed a positive effect.

The discrepancy between the findings of the present study and previous reports is likely related to the different nature of HCSL. In the current study, hydrolyzed corn steep liquor was evaluated for the first time, whereas previous investigations mainly focused on conventional CSL. The hydrolysis process may increase the concentration of soluble compounds, short peptides, organic acids, and fermentation metabolites, thereby altering the physicochemical characteristics of the product compared with conventional CSL (Guo et al., 2026). In the present study, HCSL showed distinct nutritional characteristics compared with traditional CSL (Ullah et al., 2018). Its amino acid profile was more balanced than that of traditional CSL, with higher contents of key essential amino acids such as lysine and sulfur-containing amino acids, and the proportion of each essential amino acid was more consistent with the nutritional requirements of broilers. Consequently, broiler chickens may have responded differently to HCSL than to untreated CSL. One of the major findings of the present study was the significant reduction in crude protein digestibility in the HCSL-10 treatment, which may explain the reduced BWG and impaired FCR observed in this group. Reduced protein digestibility may have increased the flow of undigested protein into the lower intestinal tract, thereby providing a favorable substrate for the proliferation of opportunistic bacteria (Apajalahti and Vienola, 2016). The intestinal morphology results further support this explanation, as dietary inclusion of 10% HCSL reduced villus height and villus surface area in the jejunum. Reduced villus height and surface area are generally associated with lower nutrient absorption capacity and impaired intestinal health (Wang et al., 2025). Under such conditions, a greater proportion of nutrients and energy may be diverted toward maintenance and regeneration of the intestinal epithelium rather than growth, ultimately leading to poorer growth performance (Hollemans et al., 2020). In addition, alterations in the ileal microbial population may have contributed to the observed decline in performance. The increased populations of E. coli and coliform bacteria, together with the reduced *Lactobacillus* population in the 7.5% and 10% HCSL treatments, suggest that high levels of HCSL disrupted intestinal microbial balance. Previous studies have also suggested that excessive inclusion of CSL may lead to excessive acidification of the gastrointestinal tract due to the high concentration of fermentable compounds and or lactic acid, thereby disturbing microbial and nutritional balance (Ebrahimpour et al., 2022; Hamidifard et al., 2023; Chukwudi et al., 2025). Although gastrointestinal pH and organic acid concentrations were not measured in the present study, it is possible that part of the negative effects observed at high HCSL inclusion levels was associated with changes in the intestinal environment. Because excessive concentrations of fermentation-derived organic acids have been reported to disrupt digestive enzyme activity and intestinal physiological balance (Sugiharto and Ranjitkar, 2019; Nourmohammadi et al., 2010; Chukwudi et al., 2025). The reduction in FI observed in the 10% HCSL treatment was likely associated with several factors. One of the main reasons may have been reduced diet palatability due to the high concentration of fermentative compounds, organic acids, and soluble substances present in HCSL, which could have negatively affected the taste and odor of the diet (Jha et al., 2019; Katu et al., 2025). Furthermore, the findings of the present study, including reduced villi height and increased opportunistic bacterial populations at high HCSL levels, suggest that impaired gut health and microbial imbalance may also lead to metabolic stress that contributes to reduced FI.

### Nutrient digestibility

The results of the present study showed that different levels of HCSL only affected crude protein digestibility, such that the inclusion of 10% HCSL significantly reduced protein digestibility compared with the control, 2.5%, and 5% HCSL treatments, whereas ash, calcium, and phosphorus digestibility were not affected by the experimental treatments. These findings indicate that the negative effects of high HCSL levels were more closely related to protein metabolism and utilization rather than mineral utilization. Moreover, the absence of negative effects at the 2.5% and 5% inclusion levels suggests that these levels of HCSL may be used as part of a soybean meal replacement strategy in broiler diets without impairing nutrient digestibility. The findings of the present study are inconsistent with a considerable number of previous reports regarding the use of CSL in poultry nutrition, as most studies have indicated that moderate dietary levels of CSL can improve nutrient digestibility, particularly protein digestibility, or at least have no detrimental effects (Ullah et al., 2018; Lee et al., 2025). However, in agreement with the results of the present study, Tauqir et al. (2022) reported that the use of a mixture of CSL and enzyme (50:50 ratio) at levels of 9 and 12% reduced crude protein and dry matter digestibility. It has been reported that the presence of soluble compounds, small peptides, free amino acids, and B vitamins in CSL may increase digestive enzyme activity and nutrient utilization (Sarwar and Khan, 2004; Azizi‐Shotorkhoft et al., 2016). Furthermore, it has been suggested that the fermentation process associated with CSL may partially degrade complex feed components, thereby improving nutrient accessibility to digestive enzymes (Qamar et al., 2015). However, most previous studies evaluated conventional CSL, and very limited information is available regarding hydrolyzed CSL products.

The reduction in crude protein digestibility observed in the 10% HCSL treatment was likely associated with several physiological and nutritional mechanisms. First, the hydrolysis process may have increased the concentration of soluble nitrogenous compounds, short peptides, and non-protein nitrogen in HCSL (Alvarez et al., 2012; Rajarathnam et al., 2016). At high inclusion levels, these compounds may disrupt amino acid absorption balance in the intestine and reduce nitrogen utilization efficiency (Zhang et al., 2007). It has been reported that imbalances in digestible amino acid profiles can decrease protein synthesis efficiency and increase nitrogen excretion (Selle et al., 2023; Nanto-Hara et al., 2026). A second possible explanation, although not directly measured in this study, could be the potential for excessive acidification of the gastrointestinal tract following high HCSL inclusion. Previous studies on CSL have suggested that, due to its high content of organic acids and fermentation-derived compounds, this ingredient may lower gastrointestinal pH (Hamidifard et al., 2023; Lee et al., 2025). While it is generally understood that a moderate reduction in pH may suppress certain pathogenic bacteria, it is hypothesized that excessive acidification could impair the activity of digestive enzymes, particularly proteolytic enzymes, thereby potentially reducing protein digestibility (Jha et al., 2019; Chukwudi et al., 2025). Furthermore, the decrease in villus height and villus surface area in the HCSL-10 treatment indicates reduced absorptive surface area and impaired intestinal epithelial function (Yamauchi, 2002). It has been reported that shortening of intestinal villi decreases nutrient contact with the absorptive surface, thereby reducing digestion and absorption efficiency, particularly for proteins (Li et al., 2020; Rysman et al., 2023). It is possible that the distinct characteristics of HCSL resulting from the hydrolysis process, together with hypothetical excessive gastrointestinal acidification, imbalance of nitrogenous compounds, unfavorable intestinal morphological changes, and disruption of microbial populations, were among the factors contributing to this response. These findings suggest that although HCSL has potential as an alternative feed ingredient, excessive inclusion levels may limit protein digestion and nutrient utilization efficiency.

### Bone quality

The results of the present study showed that high levels of HCSL in the diet negatively affected bone mineralization and bone strength, while lower levels, especially 2.5% and 5%, did not have adverse effects on bone quality. Although ash, calcium, and phosphorus digestibility were not statistically affected by the experimental treatments, the numerical reduction in ash and calcium digestibility observed in the 10% HCSL treatment may partially explain the decline in bone quality in this group. Even slight reductions in mineral absorption, particularly calcium, during the rapid growth phase of broiler chickens can impair bone mineralization and mechanical strength (Hakami et al., 2022; Han et al., 2025; Muñoz et al., 2025). Since approximately 99% of body calcium is stored in bone tissue, any disruption in calcium absorption or metabolism may reduce bone ash content and weaken bone structure (Naeem, 2025). Furthermore, the reduction in crude protein digestibility observed in the HCSL-10 treatment may also have contributed to the deterioration in bone quality (Kędzia et al., 2023). The organic matrix of bone is composed mainly of collagen protein, and reduced availability of digestible amino acids may limit bone matrix synthesis (Kareem et al., 2025). Consequently, impaired formation of the organic matrix may negatively affect mineral deposition within the bone (Naeem, 2025). Previous studies have shown that deficiencies in digestible protein or imbalances in amino acid supply can reduce bone growth and mechanical resistance in poultry (Martin et al., 2020; Lv et al., 2022; Xie et al., 2025). In addition, CSL and its derivatives generally contain high concentrations of minerals, soluble compounds, and non-protein nitrogen (Chang et al., 2025), and excessive inclusion levels may disrupt the balance among dietary energy, protein, and minerals. Such imbalance may negatively influence bone metabolism and the mineralization process (Liu et al., 2024).

### Litter and footpad health

Inclusion of 10% HCSL increased litter pH and litter ammoniacal nitrogen concentration. Furthermore, increasing dietary HCSL levels reduced the frequency of footpad dermatitis score 0, whereas the frequency of more severe lesions, particularly scores 3 and 4, increased in the 7.5% and 10% HCSL treatments. One of the main reasons for the increased litter ammoniacal nitrogen observed in the high-HCSL treatments was likely the reduction in protein digestibility and utilization. In the present study, crude protein digestibility decreased in the 10% HCSL treatment, which may have increased nitrogen excretion through the feces. Increased excretion of nitrogenous compounds, particularly uric nitrogen, provides a suitable substrate for microbial ammonia production in the litter (Such et al., 2023; Zangoli et al., 2026). Previous studies have similarly reported that any reduction in dietary protein utilization may increase litter ammoniacal nitrogen concentration (Van Emous et al., 2019; Brink et al., 2022b). The increase in litter pH observed in the HCSL-10 treatment was also likely associated with elevated ammonia production, as it is hypothesized that the degradation of nitrogenous compounds and subsequent ammonia release may increase litter alkalinity (Agyarko-Mintah et al., 2017). Previous reports have demonstrated that elevated litter pH can intensify ammonia volatilization and reduce poultry house air quality (Choi et al., 2008; Mowrer et al., 2014). Under such conditions, continuous contact of the footpad skin with alkaline litter containing high ammonia concentrations may result in tissue damage and the development of footpad dermatitis (Youssef et al., 2011; Payne et al., 2025). Although litter dry matter was not affected by the experimental treatments, the results suggest that chemical alterations in the litter, particularly increased ammoniacal nitrogen and pH, might have played an important role in increasing the severity of footpad lesions (Martins et al., 2013). This finding is consistent with previous studies indicating that footpad dermatitis is not solely dependent on litter moisture, and that litter chemical composition, especially ammonia concentration, is also a major contributing factor to skin damage (Youssef et al., 2011).

### Jejunum morphology

Morphology findings indicate that low levels of HCSL were able to maintain normal intestinal structure, whereas higher inclusion levels, particularly 10%, exerted adverse effects on intestinal health and absorptive capacity. Greater villus height and higher villus height to crypt depth ratio are generally considered indicators of improved intestinal health and enhanced nutrient absorption capacity (Wang et al., 2025). Longer villi provide a larger surface area for nutrient absorption, whereas reductions in these parameters are commonly associated with impaired epithelial renewal or increased stress on the intestinal mucosa (Nguyen et al., 2021; Alagbe et al., 2026). Furthermore, the reduction in villus surface area observed in the HCSL-10 treatment suggests that nutrient absorption capacity was likely compromised in this group, which may partly explain the reduced growth performance and protein digestibility observed in birds fed high levels of HCSL. The findings of the present study partially differ from previous reports regarding the use of CSL in poultry nutrition. Many studies have demonstrated that moderate inclusion levels of CSL or similar fermented products may improve intestinal morphology, which has been attributed to the presence of bioactive compounds, B vitamins, amino acids, and fermentation-derived metabolites (Hamidifard et al., 2023; Lee et al., 2025). It is possible that the hydrolysis process alters the chemical characteristics of HCSL and increases the concentration of soluble compounds and organic acids, which at high inclusion levels may negatively affect the intestinal epithelium (Sugiharto and Ranjitkar, 2019). Previous studies have indicated that high concentrations of organic acids and fermentation-derived compounds may disrupt the physiological balance of the intestinal mucosa when consumed excessively (Andreopoulou et al., 2014; Katu et al., 2025). In addition, the reduction in crude protein digestibility observed in the HCSL-10 treatment may have been associated with the observed intestinal morphological changes. Reduced villus height and absorptive surface area may limit nutrient contact with the intestinal epithelium and consequently reduce digestive and absorptive efficiency (Azizi et al., 2026). Furthermore, under conditions of intestinal structural impairment, a proportion of dietary nutrients and energy may be diverted toward intestinal tissue repair and maintenance, thereby negatively affecting growth performance and feed efficiency (Oni et al., 2025).

### Specific ileal microbiota counts

Dietary inclusion of 7.5% and 10% HCSL increased the population of *E. coli* and reduced the population of *Lactobacillus*, whereas the 2.5% and 5% HCSL treatments showed lower *coliform* populations compared with the control and higher HCSL levels. These findings indicate that low levels of HCSL may partially improve intestinal microbial balance, whereas higher inclusion levels, particularly 10%, disrupt the intestinal microbial ecosystem. The reduction in *coliform* populations observed in the 2.5% and 5% HCSL treatments was likely associated with the presence of fermentation-derived compounds, organic acids, and certain bioactive substances in HCSL. Previous studies on CSL and similar fermented products have shown that moderate levels of these compounds may reduce intestinal pH and inhibit the growth of pathogenic bacteria, thereby creating favorable conditions for beneficial microorganisms (Ebrahimpour et al., 2022). In addition, some fermentation metabolites may exert prebiotic-like effects and contribute to stabilization of beneficial microbial populations. CSL contains 22% lactic acid and is low in acidity (pH = 2.4) (Ullah et al., 2018). It is hypothesized that organic acids, such as lactic acid, can pass through the bacterial cell membrane in an undissociated form and, after dissociating, produce hydrogen ions inside the cell, which could lower intracellular pH. Under such conditions, pH-sensitive microorganisms, such as *coliforms*, must expend energy to transport hydrogen ions out of the cell. The resulting anionic accumulation and intracellular acidification might also interfere with the synthesis of DNA and cellular proteins, potentially reducing the proliferation and population of this group of microorganisms in the intestine. On the other hand, beneficial and pH-insensitive microorganisms, such as lactic acid bacteria, are reported to be capable of maintaining intracellular pH homeostasis under acidic conditions (Adil et al., 2011). This mechanism could potentially explain the reduced coliform counts observed at lower HCSL inclusion levels. However, increasing dietary HCSL levels to 7.5% and 10% produced the opposite effect, resulting in increased *E. coli* populations and reduced *Lactobacillus* counts. These findings suggest that excessive inclusion of HCSL might have disrupted the normal microbial balance of the intestine (Sugiharto and Ranjitkar, 2019). The reduction in crude protein digestibility observed in the HCSL-10 treatment may also have contributed to the increased populations of opportunistic bacteria (Elling-Staats et al., 2022). When dietary protein is not adequately digested and absorbed, larger amounts of nitrogenous compounds reach the lower gastrointestinal tract and serve as substrates for the growth of proteolytic and opportunistic bacteria (Elling-Staats et al., 2023). Previous studies have shown that increased levels of undigested protein in the intestine may stimulate the growth of *E. coli* and other *coliform* bacteria, thereby disrupting intestinal microbial balance (Kim et al., 2022). This may partly explain the differences between the findings of the present study and some previous reports describing positive effects of conventional CSL on intestinal microbial populations.

## Conclusion

In conclusion, the findings of the present study demonstrated that the response of broiler chickens to dietary HCSL was highly dependent on inclusion level. Low dietary levels of HCSL (2.5 and 5%) showed the potential to be used as a partial alternative feed ingredient without adverse effects on growth performance, nutrient utilization, intestinal health, bone quality, litter condition, or microbial balance. However, excessive inclusion levels (7.5 and 10%) negatively affected several physiological and productive parameters, likely due to alterations in gastrointestinal environment, nutrient utilization efficiency, and intestinal microbial homeostasis. Therefore, although HCSL may represent a promising alternative feed resource in broiler nutrition, careful optimization of dietary inclusion levels is essential to avoid detrimental effects on bird health and performance.

## Disclosures

The authors declare that this research was conducted in the absence of any commercial or financial relationships that could be construed as a potential conflict of interest. No funding was received from any feed additive manufacturer, ingredient supplier, or any other commercial entity with a financial interest in hydrolyzed corn steep liquor or related feed ingredients. The study design, data collection, statistical analysis, interpretation of results, and decision to publish were carried out independently by the authors, without any influence from external commercial parties. All authors have read and approved the final manuscript and affirm that the findings reported herein reflect objective scientific inquiry free from any bias or undisclosed competing interests.
